# Streamlined quantitative analysis of histone modification abundance at nucleosome-scale resolution with siQ-ChIP version 2.0

**DOI:** 10.1038/s41598-023-34430-2

**Published:** 2023-05-09

**Authors:** Bradley M. Dickson, Ariana Kupai, Robert M. Vaughan, Scott B. Rothbart

**Affiliations:** 1 Department of Epigenetics, Van Andel Institute, Grand Rapids, MI 49503 USA; 2 Department of Pediatrics and Human Development, Michigan State University, Grand Rapids, MI USA

**Keywords:** Biochemistry, Biological techniques, Computational biology and bioinformatics

## Abstract

We recently introduced an absolute and physical quantitative scale for chromatin immunoprecipitation followed by sequencing (ChIP-seq). The scale itself was determined directly from measurements routinely made on sequencing samples without additional reagents or spike-ins. We called this approach sans spike-in quantitative ChIP, or siQ-ChIP. Herein, we extend those results in several ways. First, we simplified the calculations defining the quantitative scale, reducing practitioner burden. Second, we reveal a normalization constraint implied by the quantitative scale and introduce a new scheme for generating ‘tracks’. The constraint requires that tracks are probability distributions so that quantified ChIP-seq is analogous to a mass distribution. Third, we introduce some whole-genome analyses that allow us, for example, to project the IP mass (immunoprecipitated mass) onto the genome to evaluate how much of any genomic interval was captured in the IP. We applied siQ-ChIP to p300/CBP inhibition and compare our results to those of others. We detail how the same data-level observations are misinterpreted in the literature when tracks are not understood as probability densities and are compared without correct quantitative scaling, and we offer new interpretations of p300/CBP inhibition outcomes.

## Introduction

The chromatin immunoprecipitation (ChIP) method was introduced in the 1980’s to analyze DNA-protein interactions at specific genomic loci in prokaryotic and eukaryotic cells^1,2^. In general, the method involves in-cell fixation of chromatin-associated proteins to DNA, chromatin extraction and fragmentation, IP of chromatin fragments with antibodies specific to the target protein or post-translational modification (PTM) state, DNA isolation, and analysis of enriched fragments by hybridization, amplification, and sequencing methods. With few modifications to this method, and recent adaptation for compatibility with high-throughput sequencing (seq), ChIP-seq is now widely deployed for studying DNA-associated protein location across genomes^3^.

There is a perception that ChIP-seq is not a quantitative method^4^. As such, the chromatin community has developed modifications to ChIP-seq protocols involving the introduction of spike-in reagents at various stages of sample preparation to establish relative scales^5–9^. The goal of these signal normalization approaches is to enable direct comparison of ChIP-seq results across samples and provide an accurate means of determining, for example, how cellular perturbations impact the distribution of histone PTMs and chromatin-associated proteins across genomes. However, these relative scales are not defined in terms of absolute quantities or units. Moreover, a lack of method standardization and bookkeeping practice makes it impossible to directly compare ChIP-seq datasets from experiment to experiment within the same lab, across different labs, and from datasets compiled as part of large-scale consortium initiatives like the EnCODE Project^10^ *even when spike-ins are used*^11^. The lack of reporting is particularly problematic since the distribution of antibody capture efficiency across the genome is a function of IP conditions^12^. If the reaction conditions are different enough to change antibody distribution, then clearly no global normalizer (or spike-in) can correct for this.

We recently introduced sans spike-in quantitative ChIP-seq^11^ (siQ-ChIP), a method that emerged from the concept that ChIP-seq is itself inherently quantitative on an absolute scale by virtue of the equilibrium binding reaction in the IP of chromatin fragments. The theoretical model of this equilibrium binding reaction, as introduced in our prior work, proposed that the captured IP mass would follow a sigmoidal isotherm if the reaction was governed by classical mass conservation laws. If we could map the number of sequenced fragments into the total number of fragments contained in the IP product, then we could obtain a quantitative scale through connection to the isotherm. Cellular perturbations that change protein or PTM presentations would emerge as changes in position on the isotherm, and would thus be directly quantitatively comparable.

Informed by continued theoretical analysis and experimental practice, we report further development of the proportionality constant, $\alpha$, that is needed to compute the siQ-ChIP quantitative scale. The improved expression for $\alpha$ is simple to understand, simple to evaluate, and importantly, results in values that are identical between the old and new expression. This new expression highlights a novel normalization constraint, ignored by the community, related to how sequenced fragments are aggregated into visual representations. This constraint can impact global track shape and has implications on how tracks should be interpreted. We discuss some published misinterpretations as examples. We also introduce novel modes of automated whole-genome analysis that can be used to easily visualize and compare outcomes of cellular perturbation on the distribution and abundance of histone PTMs as measured by siQ-ChIP.

## Results and discussion

In this section, we derive a simplified expression for the proportionality constant $\alpha$ that enables quantitative ChIP-seq and we introduce some consequences for track building. This new expression is more intuitive to understand, easier to evaluate, and more accurate to sequencing outcomes than the previous expression. While values derived from old and new expressions are consistent, the new expression demonstrates a clear and explicit dependence on paired-end sequencing.

Two distinct derivations of $\alpha$ are presented in this paper. Below, we show how the original expression for $\alpha$ can be explicitly reduced to a simplified expression. We present this approach here to stress the consistency between the new and old^11^ expressions. However, we also describe a more intuition-driven derivation in Supplementary Information (SI). This derivation starts from the mass-conservation laws that describe the IP reaction and develops an expression for the total concentration of antibody-bound chromatin fragments, $S^b$, which is the sum of all epitope species that interact with the antibody. The final siQ-ChIP scaled sequencing track is given by projecting $S^b/S^t$ onto the genome, where $S^t$ is the total concentration of all species in the sample chromatin. Thus, the siQ-ChIP scale is literally the IP reaction efficiency. In the SI we show how this scale is obtained by a simple unit conversion applied to the commonly measured IP mass and input mass. This provides an intuitive, but exact, description of the siQ-ChIP scale and shows how everything is coupled to the mass-conservation laws.

Recall that the total bound concentration of chromatin will follow a sigmoidal binding isotherm^11^. The isotherm itself can be constructed by performing multiple IPs, each at increasing amounts of antibody with fixed chromatin concentration (or vice versa), and plotting the captured DNA mass as a function of antibody used. This isotherm is the central landmark for siQ-ChIP because it establishes control over the reagents, defines the quantitative scale, and generates a reproducible target object. Different points along the isotherm can also be sequenced to better understand antibody dynamics^12^. For each point on the isotherm, a value of the siQ-ChIP normalizer can be computed, and the resulting sequencing data can be quantified.

### A simplified expression for the proportionality constant $\alpha$

In our previous work^11^, we built the inherent ChIP-seq quantitative scale by first noting that the total number of reads available in a given IP can be written as

1 $$\begin{aligned} R=\frac{\hat{R}}{\mathscr {F}^l\,2^c\,\rho \,\mathscr {F}} \end{aligned}$$

where $\hat{R}$ is the sequenced depth of the IP and *R* is the total possible depth if the full IP mass were sequenced. $\mathscr {F}^l$ is the fraction of library sequenced, $\rho$ is the library concentration divided by the theoretical library concentration (what we call the *library efficiency*), and $\mathscr {F}$ is the fraction of IP’d material taken into library prep. $2^c$ accounts for particle doublings encountered during amplification and adaptor ligation. (See SI-Table 1 for a full summary of symbols.)

Combining Eq. (1) with the analogous expression for input, and taking the difference in volumes for IP and input into consideration, one obtains the quantitative ChIP-seq scaling factor $\alpha$ as^11^

2 $$\begin{aligned} \alpha = \frac{v_{\textrm{in}}}{V-v_{\textrm{in}}}\frac{\rho _{\textrm{in}}}{\rho }\frac{\mathscr {F}^l_{\textrm{in}}}{\mathscr {F}^l}\frac{\mathscr {F}_{\textrm{in}}}{\mathscr {F}} \end{aligned}$$

Here, $v_{\textrm{in}}$ is the input sample volume and $V-v_{\textrm{in}}$ is the IP reaction volume.

The expression for $\alpha$ can be simplified considerably by writing all the factors of $\alpha$ in their base units and cancelling as many contributions as possible. Equation (1) can be reduced to (writing all the terms in mass units)

3 $$\begin{aligned} R=\frac{\hat{R}}{\frac{m_{\textrm{loaded}}}{m_l}2^c\frac{m_l}{2^cm_\mathrm{to\_lib}}\frac{m_{\mathrm{to\_lib}}}{m_{\textrm{IP}}}}= m_{\textrm{IP}}\frac{\hat{R}}{m_{\textrm{loaded}}} \end{aligned}$$

where $m_{\mathrm{to\_lib}}$ is the mass taken into library prep, $m_{\textrm{loaded}}$ is the mass loaded onto the sequencer, and $m_{\textrm{IP}}$ is the full IP mass. The total possible reads that can be extracted from an IP is expressed as the product of the IP mass, $m_{\textrm{IP}}$, and a reads per unit mass conversion factor $\hat{R}/m_{\textrm{loaded}}$. Alternatively, the unitless ratio $m_{\textrm{IP}}/m_{\textrm{loaded}}$ scales the actual depth $\hat{R}$ to the total possible depth *R*.

Using Eq. (3), and the analogous result for input reads, we can rewrite $\alpha$ in a more intuitive and simplified way, where

4 $$\begin{aligned} \alpha =\frac{v_{\textrm{in}}}{V-v_{\textrm{in}}}\frac{m_{\textrm{IP}}}{m_{\textrm{in}}}\frac{m_{\textrm{loaded,in}}}{m_{\textrm{loaded}}} \end{aligned}$$

Finally, the fraction $\frac{m_{\textrm{loaded,in}}}{m_{\textrm{loaded}}}$ can be reinterpreted through the following observation. The total sequencing reads generated by a single flow-cell are commonly split among several samples. Loading a multiplexed flow-cell can be idealized as: Each sample is standardized to the same molarity, then different volumes are taken from each sample and pooled. The volume fraction of each sample now corresponds to the fraction of total particles that come from that sample. In this circumstance, the fraction of the flow-cell’s reads that will be consumed by each sample is given by its volume fraction in the pool. The expectation is that the conversion from moles of chromatin fragments to sequencer reads is constant, $\frac{\hat{R}}{m_{\textrm{loaded}}/(660\cdot L)}\sim \frac{\hat{R}_{\textrm{in}}}{m_{\textrm{loaded,in}}/(660\cdot L_{\textrm{in}})}$. Here, 660 is the average molecular weight of a DNA base pair (*g*/*mol*/*bp*), and $L_{\textrm{in}}$ and *L* are the average fragment lengths for input and IP respectively. These are library fragment lengths, reported in our case by a Bioanalyzer. The moles to reads relationship between input and IP can be rearranged and substituted into $\alpha$ to produce

5 $$\begin{aligned} \alpha =\frac{v_{\textrm{in}}}{V-v_{\textrm{in}}}\frac{m_{\textrm{IP}}}{m_{\textrm{in}}}\frac{\hat{R}_{\textrm{in}}}{\hat{R}}\frac{L_{\textrm{in}}}{L} \end{aligned}$$

**Figure 1:**
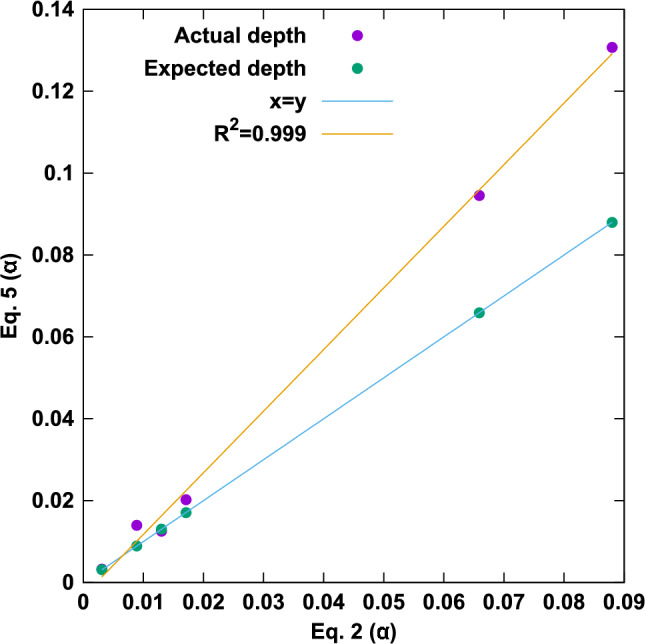
Comparison of the new and old expressions for $\alpha$. Direct comparison of $\alpha$ evaluated by Eqs. (2) and (5) where depths are taken as actual (observed) or expected (requested). The orange line is $m*x+b$ with $m=1.505$ and $b=-0.0033$.

The symbols $\hat{R}$ and $\hat{R}_{\textrm{in}}$ represent the number of sequencing reads (or fragments) generated by IP and input, respectively. We have cancelled the factors of $660\,g/mol/bp$ in Eq. (5). Figure 1 shows the correspondence between Eqs. (2) and (5) for the data reported in this paper. The new $\alpha$ can be evaluated for expected depth or actual depth, whereas the previous form explicitly used mass loaded into sequencing and is therefore limited to expected depth. It is common to find errors in pipetted volumes or in mass measurements during the sequencing process, and these errors result in the number of actual sequenced fragments being different from the expected number of sequenced fragments. As we show below, it is critical that the actual number of fragments be used in analysis (not the expected number) to prevent normalization errors. Figure 1 clearly demonstrates the correspondence of Eqs. (2) and (5) when expected depth is used. In summary, the new $\alpha$ is simpler to understand, easier to evaluate, and more accurate to sequencing outcomes because we can make use of the obtained depth rather than the requested depth.

**Figure 2:**
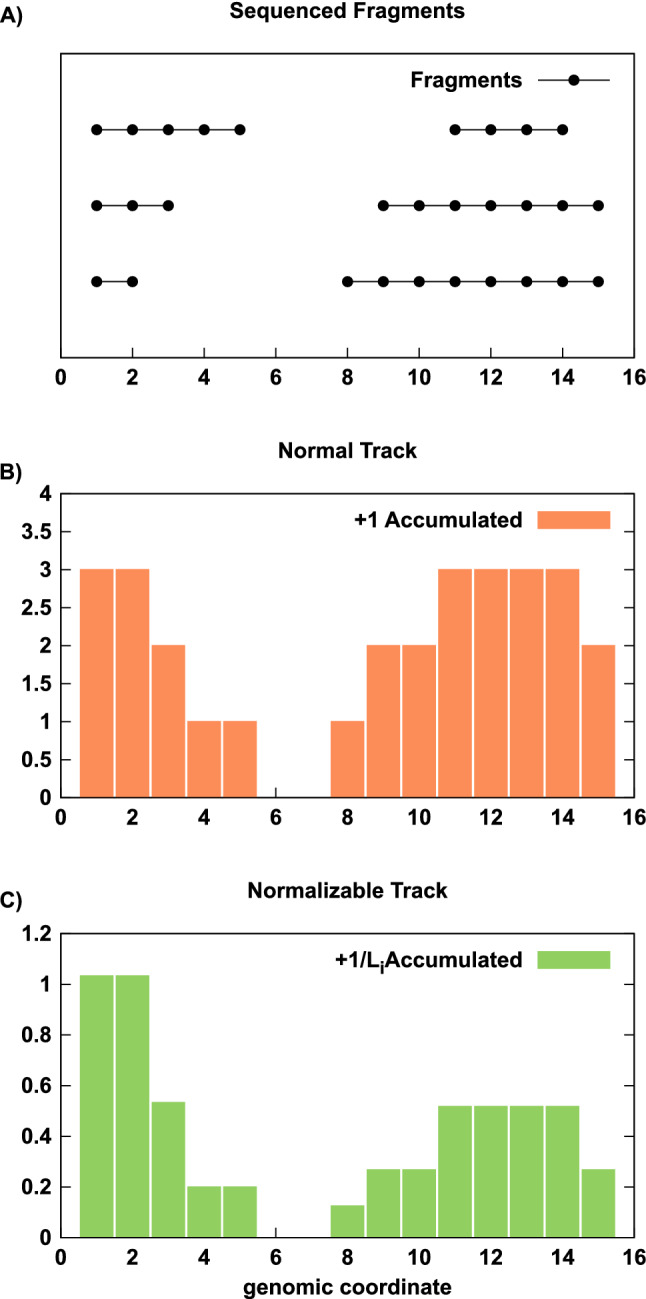
Illustration of normalized coverage: Example track builds from 6 sequenced fragments. Here we illustrate the impacts of counting scheme on the shape of the final track. (**A**) and accumulating $+1$ (**B**) or $+1/L_i$ (**C**) when counting fragments at each genomic position.

### Normalized coverage

The final, quantified siQ-ChIP signal (or track) is $S^b(x)/S^t(x)$, which is an expression of the IP reaction efficiency on the genomic interval *x*. This quantity is obtained as $\alpha \frac{f_{\textrm{IP}}(x)}{f_{\textrm{in}}(x)}$, where *f*(*x*) is a track for either the IP or input sequencing. The specific definition of *f*(*x*) is the main focus of this section. In SI we show a derivation of $\alpha$ that leads to an intuitive representation of the siQ track as $\frac{c_{\textrm{IP}}f_{\textrm{IP}}(x)}{c_{\textrm{in}}f_{\textrm{in}}(x)}$, where the coefficients *c* convert the IP or input mass into concentration units and normalize the respective *f*(*x*). The tracks *f*(*x*) that enable the projection to genome, in a way consistent with the structure of $\alpha$, must be defined precisely, as discussed next.

The resulting, more intuitive, perspective on $\alpha$ comes by viewing it as the ratio of two factors $\alpha =\frac{c_{\textrm{IP}}}{c_{\textrm{in}}}$ with

6 $$\begin{aligned} c_{\textrm{IP}} & = \frac{m_{\textrm{IP}}}{660L(V-v_{\textrm{in}})}\frac{1}{\hat{R}}\\ c_{\textrm{in}}& = \frac{m_{\textrm{in}}}{660L_{\textrm{in}}v_{\textrm{in}}}\frac{1}{\hat{R}_{\textrm{in}}} \end{aligned}$$

Each of these coefficients is written as the product of two quotients. The first expresses the IP or input mass as a concentration by direct units conversion. The second is a normalization factor pertaining to depth. Therefore, if *f*(*x*) is a browser track of the IP sequenced fragments, which is just a histogram of fragments intersecting base pair *x*, then $c_{\textrm{IP}}f(x)$ is the concentration of DNA that overlaps *x* that was bound in the IP reaction. This projection of bulk concentration to genomic location is valid if, and only if, $\frac{1}{\hat{R}}\sum _x f(x)=1$. When the track *f*(*x*) is built, each fragment can be counted only once so that $1/\hat{R}$ normalizes *f*(*x*). We suggest that *f*(*x*) be referred to as the ‘normalized coverage’.

**Figure 3:**
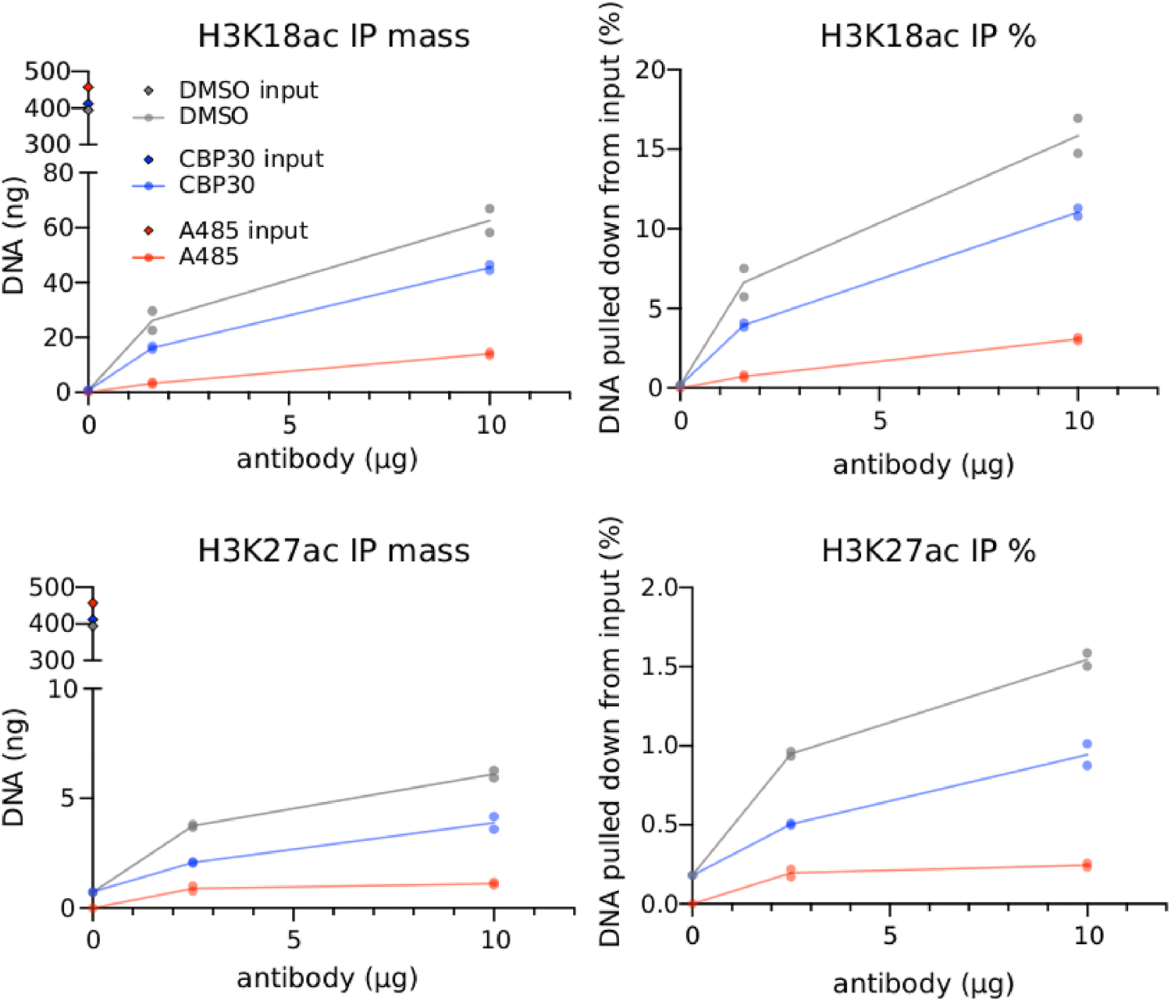
Isotherms of ChIP mass capture. H3K18ac (top) and H3K27ac (bottom) ChIP antibody titrations for HeLa chromatin extracted following DMSO, CBP30, or A485 treatment. Data from two technicalreplicates are reported as total mass (nanograms left) and percent input (right).

**Figure 4:**
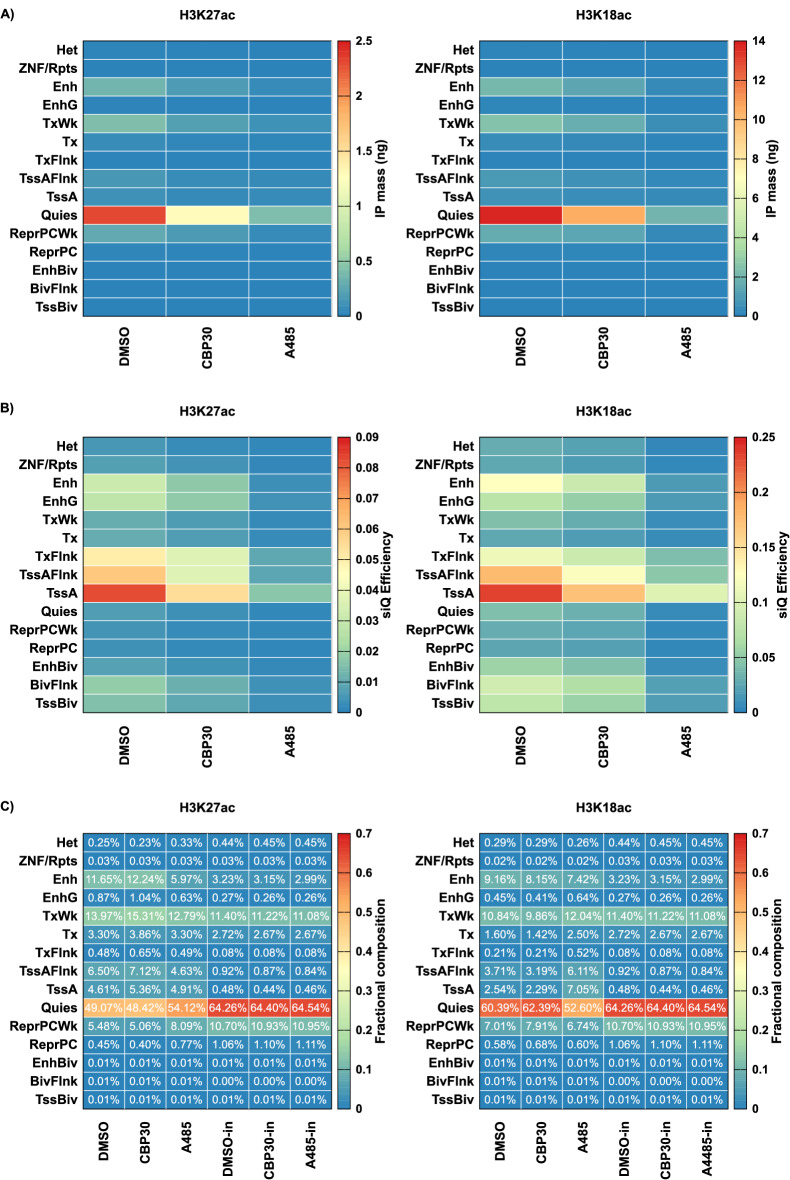
Analysis of sequenced fragment distributions. The 15-state model from the Roadmap Epigenomics Consortium was used to generate: (**A**) mass of IPs projected onto annotation after DMSO, CBP30, or A485 treatment. (**B**) siQ-ChIP capture efficiency. (**C**) the fractional composition of IPs.

**Figure 5:**
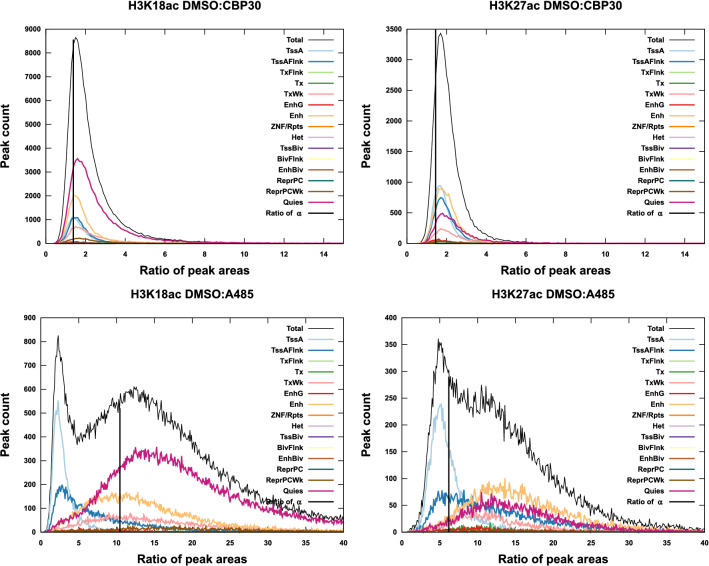
The distributions of peak responses to p300/CBP inhibition, $\mu (r)$, **in sequencing peaks.** The distributions of peak responses to p300/CBP inhibition, $\mu (r)$, in sequencing peaks. The response distribution is shown as a total (black) and decomposed into contributions by annotation. The vertical black line indicates the ratio of $\alpha$ for the two tracks being compared.

The standard process of building tracks for use in a browser yields tracks that do not satisfy this normalization constraint. If, for example, the *i*-th sequenced fragment accumulates a $+1$ at every base pair that it intersects, then the *i*-th fragment is over-counted $L_i$ times, with $L_i$ the length of the fragment in base pairs.

Accumulating $+1/L_i$ at each intersected base pair, instead of $+1$, resolves the issue of overcounting entirely. A track built this way is a proper histogram and is normalized by the number of observations that went into the histogram, $\hat{R}$ for an IP and $\hat{R}_{\textrm{in}}$ for input, and is suitable for genome browsers. In this scheme, each base pair in a fragment is equally weighted, just like when $+1$ is accumulated. However, different fragments are not equally weighted unless they have the same length. In particular, longer fragments will effectively contribute with lower weight because there is a greater uncertainty in ‘where’ the important binding event was when that fragment was captured. Note that paired-end sequencing is required to correctly determine the $L_i$.

In Fig. 2, we illustrate six sequenced fragments and show the outcome of building a track using the $+1$ or $+1/L_i$ accumulations. The set of six fragments form two ‘islands’, where fragments within an island do not overlap fragments from outside the ‘island’. When $+1/L_i$ is used, each ‘island’ of piled fragments will reflect a sum of fractions that looks like $1/L_1 + 1/L_2 + ...$. Each ‘island’, then, can be understood as having its own common denominator for the summation of the fractions. In our example, the left island has a common denominator of 30 while the right island has a common denominator of 56. The fact that islands have different common denominators allows the islands to have different final scales. For example, when the $+1$ weights are used, the left and right islands form peaks of equal height (Fig. 2B). When the $+1/L_i$ weights are used, the right island forms a shorter peak than the left island (Fig. 2C). This is because the right island is comprised of longer fragments and these fragments convey a larger uncertainty about where the peak ought to focus.

**Figure 6:**
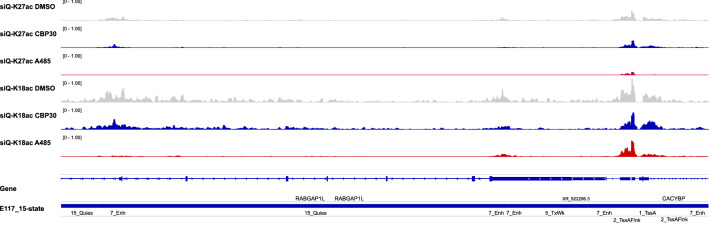
A sample browser shot illustrating the diversity of TssA and Enh responses in H3K27ac and H3K18ac quantitative ChIP-seq data. All ChIP-seq data ranges are [0, 1] for direct comparison between tracks and conditions, all windowing functions are disabled. The displayed location is a 43 kb window of the genome centered on chr1:174960844-175004779.

**Figure 7:**
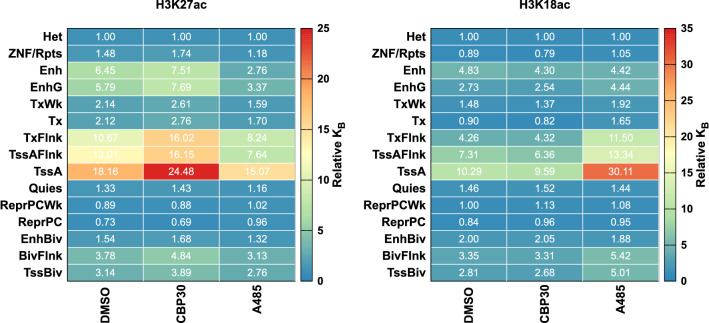
Relative binding constants. The relative binding constants between chromatin compartments or peaks within a track can be estimated by Eq.  (8). Relative binding constants are shown here with the Het annotation taken as the reference. Any annotation could be specified as a reference.

**Figure 8:**
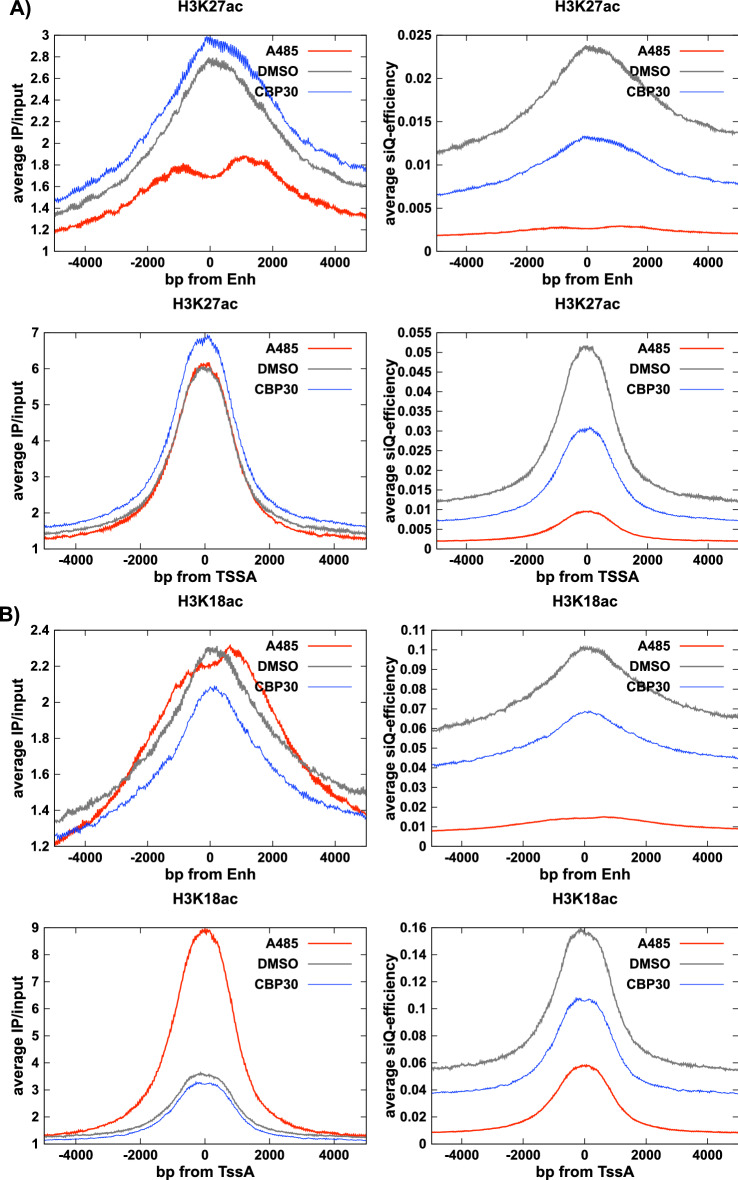
Impact of physical scaling on metaplots. Metaplots in unscaled (left) and siQ-scaled (right) units for Enh and TssA annotations captured by (**A**) H3K27ac or (**B**) H3K18ac antibodies.

The scaling of the sequencing tracks by $\alpha$ (or $c_{\textrm{IP}}$, etc) can only be correctly interpreted as a projection of the physical IP outcome onto genomic position if $f(x)/\hat{R}$ is a normalized probability distribution. This constraint on building *f*(*x*) is a key insight that has not been provided by any other analysis of ChIP-seq, yet it is critical for preservation of physical scale, and as we have just demonstrated, can impact track shape across the genome. In light of this constraint, arbitrary scaling rules like RPKM (reads per kilobase of million mapped) become unnecessary.

Moreover, any material quantity can be projected onto the genome with a correctly assembled track. For example the IP mass itself can be projected onto genomic coordinates, $m_{\textrm{IP}}f(x)/\hat{R}$, allowing one to compute the mass contributed to the IP from any genomic interval. It is worth noting that, formally, quantitative ChIP-seq could be defined through this projection of IP mass in order to avoid sequencing an input sample. Two IP mass projections could be directly compared if the IP conditions were matched as siQ-ChIP requires, although this forces an uncontrolled assumption on the invariance of input samples at the genomic level.

Most importantly, even if siQ-ChIP is not used the $+1/L_i$ counting scheme can be easily implemented in any track building software. With the tracks being constructed as probability densities, interpretation of tracks will be restricted to the sense of distribution (not PTM level) and would even be amenable to the above described IP-mass projection scheme. This would offer at least some, very crude level of quantification to all ChIP-seq experiments.

To summarize, the quantity $c_{\textrm{IP}}f(x)$ is an estimate of the concentration of chromatin bound in the IP that originated from position *x*. Likewise, $c_{\textrm{in}}f_{\textrm{in}}(x)$ is an estimate of the total concentration of chromatin in the input that originated from position *x*. The siQ-ChIP “track” in quantitative units is given by $\alpha f(x)/f_{\textrm{in}}(x) = \frac{c_{\textrm{IP}}f(x)}{c_{\textrm{in}}f_{\textrm{in}}(x)}$ and is an estimate of the IP binding efficiency at position *x*, *i.e.,* the fraction of chromatin originating from *x* that is bound in IP.

The above development of $\alpha$ and its dependence on average fragment length motivate some comments on our practice of chromatin fragmentation. Complex distributions of fragment length introduce error in mass-to-concentration conversions and may artificially inflate IP capture masses. We found Micrococcal Nuclease (MNase) digestion of chromatin produced narrow fragment length distributions, especially when compared to the typical outcome of sonication. Notably, and consistent with prior work^13^, MNase digestion does not lead to bias in the ability to observe heterochromatic nucleosomes with this assay protocol. Details of our protocol are available elsewhere^12^.

Finally, we note that this simplification of $\alpha$ requires the practitioner to report 6 parameters at the start of compiling siQ-ChIP data. (These parameters are the volumes, masses and average fragment lengths. The depths will be gleaned from the mapped sequencing files.) The previous form of $\alpha$ required dozens of entries to compute the same value. It is also worth noting that all of the following analyses have been automated in the current version of the siQ-ChIP software, which can be found on GitHub^14^.

### CBP/p300 inhibition via CBP30 and A485

To demonstrate the utility of siQ-ChIP with working examples, we considered the impact of inhibiting p300/CBP on acetylation at lysines 18 and 27 on histone 3 (H3K18ac and H3K27ac, respectively). We analyzed the effects of two inhibitors, CBP30^15^ and A485^16^. CBP30 targets the bromodomain of p300/CBP while A485 targets the acetyltransferase domain. This system has been recently characterized by others^17–20^, allowing a comparison of our ChIP-seq analysis to existing results. Importantly, all of the previous work reports and interprets ChIP-seq data that are not quantified in absolute terms. We therefore directly examine the role of absolute ChIP-seq quantification in interpreting observed consequences of the two modes of p300/CBP inhibition.

Central to the siQ-ChIP paradigm is the antibody:chromatin isotherm^11^. This isotherm is, in reality, a many-dimensional surface, with the coordinates in its domain being the concentration of antibody, and the concentration of each epitope. For each of our experimental contexts (CBP30 inhibition, A485 inhibition, DMSO control) we determined the antibody:chromatin isotherm by titrating antibody. The isotherms are shown in Fig. 3, where all epitope coordinates are held fixed and the antibody concentration was titrated. Because the total chromatin concentration is fixed (to within experimental ability), the change in isotherm as a function of target-epitope concentration is approximated by the changes seen in moving from DMSO to CBP30 to A485. Keep in mind that the total chromatin concentration is fixed, only the concentration of p300/CBP dependent epitopes has changed. Figure 3 informs on the full surface of the antibody:chromatin isotherm and is a useful global landmark for assessing consistency of repeat experiments. For accurate quantitation and replication, IP masses should always be reported with ChIP-seq data, along with evidence that the IP conditions were matched (*e.g.,* by reporting input masses). The IP mass can confer notions of losses or gains which can be used as a reference against the apparent changes observed in sequenced data. The power of these isotherms is that we have more than one observation on the mass at more than one antibody load. These observations can be considered for self-consistency as well as referenced against any future repeats.

Several observations come from the data presented in Fig. 3. First, H3K18ac produces substantially higher IP masses than does H3K27ac. Both the H3K18ac and H3K27ac antibodies appeared to have their isotherm inflection points to the left of the 2.5 and 1.6 µg antibody loads, evidenced by the fact that both antibodies appear to be approaching saturation — Each antibody displayed diminishing returns after an $\sim 8$ fold increase in concentration. The loads 1.6 and 2.5 µg differ between antibodies because of limiting reagent constraints. Figure 3 suggests either that H3K18ac is more abundant in HeLa chromatin than H3K27ac or that the H3K18ac antibody is binding to many off-target chromatin species. These data do not argue that the H3K27ac antibody is weaker, and thereby captures less mass, because of the change in slope from 0 to 2.5 and from 2.5 to 10 µg antibody. A weaker antibody will not simply plateau at a lower captured mass but will instead plateau only at higher antibody loads. Reports coming from mass spectrometry suggest that H3K18ac is more abundant than H3K27ac^21,22^, consistent with the isotherms in Fig. 3.

Second, the isotherms show that A485 is effective (more so than CBP30) at globally reducing levels of H3K18ac and H3K27ac with the treatment paradigm used here. At low antibody load, CBP30 produced a 1.4-fold reduction in IP mass for H3K18ac and a 1.9-fold reduction in IP mass for H3K27ac. The moderate effects of CBP30 on these PTMs is consistent with prior work^18^. Meanwhile, under the same treatment and IP conditions, A485 produced a 6.1-fold reduction in H3K18ac IP mass and a 4.9-fold reduction in H3K27ac IP mass. We carried the 1.6 and 2.5 µg antibody points into sequencing, because this is where we would predict the highest antibody specificity^11^.

Note that the isotherms also demonstrate that the IP mass is controlled by the antibody load. If the isotherm were insensitive to antibody load, for example if the isotherm were flat or if only a single IP point were collected, it would be impossible to infer that the IP mass is indeed due to action of the antibody. The following sections address analysis of the sequencing data.

### Whole genome analysis using annotations

The formal model of the IP binding process at the heart of siQ-ChIP explicitly expands chromatin into a list of all possible chromatin modification states^11^, where we call each state a species. Below we explore the possibility that genomic annotations may provide a means of grouping several distinct (but similar) species into larger classes. Each annotation is, of course, the aggregate of several species, but decomposition of the IP products into annotations turns out to be very useful nonetheless. This analysis provides a simple way to understand how the global distribution of fragments is changed by experimental perturbation.

We take the 15-state model put forward by the NIH Roadmap Epigenomics Consortium as our model for chromatin-state (and therefore epitope/species) labels^23^. To estimate the distribution of the sequenced fragments with respect to the 15 distinct annotations, let $a_i$, with $i=1,2,...,15$, indicate the *i*th annotated state. Let $f_{\textrm{IP}}(a_i)$ be the total number of IP reads that intersect any genomic interval annotated by $a_i$ (if a fragment intersects two or more, arbitrarily take the first one). Notice that $\sum _i f=\hat{R}$, just as we required above. As a novel representation of the sequencing data, the IP mass can be projected onto the annotations as $L(V-v_{\textrm{in}})660\times c_{\textrm{IP}}f_{\textrm{IP}}$. Figure 4A shows the IP masses from H3K27ac and H3K18ac projected onto the annotations, thus decomposing the total IP masses into contributions from each class of genomic annotation. Each column of Fig. 4A sums to the total mass for that experiment.

Figure 4B reports the siQ capture efficiency ($\alpha f_{\textrm{IP}}/f_{\textrm{in}}$) of H3K18ac and H3K27ac for each annotation. Notice that the largest IP mass in either H3K18ac or H3K27ac was due to the Quies annotation (Fig. 4A), but the largest capture efficiency was due to the TssA annotation for both H3K18 and H3K27 (Fig. 4B). The capture efficiency of Quies is actually small for both H3K18ac and H3K27ac. The IP masses in Fig. 4A do not report on the input composition, but the siQ capture efficiency does. This is why quantification in terms of capture efficiency is important and more meaningful than mass alone. Likewise, the mass itself does not report on *enrichment*. By looking at capture efficiency, we can see that both antibodies enrich for annotations that reflect active chromatin states. On the other hand, looking at the masses shows that a great deal of sequencing reads were consumed by Quies regions and that, surprisingly, the capture at Quies is dependent on p300/CBP activity.

Along with siQ-ChIP, we previously introduced the *fractional composition* of the IP as a way to present the distribution of IP products, and we studied this distribution through simulations to show that it can behave in some counterintuitive ways^11^. The fractional composition is identical to the distribution of fragments over the annotations, $f(a_i)/\hat{R}$. The use of annotations as a proxy for species allows us to examine the fractional composition of actual IPs, and to visualize how the distribution of IP products responded to different p300/CBP inhibition paradigms. Figure 4C shows the fractional composition for each IP and input, computed as $f(a_i)/\hat{R}$. Perhaps the most notable feature emerging from this analysis is the increase in TssA- and TssAFlnk- (active promoter flanking) associated fragments in the H3K18ac pulldown after A485 treatment. (2.8- and 1.6-fold, respectively, Fig. 4C) This increase reflects the increased probability of observing a TssA-associated fragment, when randomly selecting fragments from the IP. This increase does not indicate an increase of H3K18ac at TssA annotations, as both the capture efficiency and captured mass are down after inhibition (Fig. 4A,B). An increase in this probability could, in general, be due an increase in PTM, an unaffected local population of PTM with PTM losses in other regions, or a less affected local PTM population. The data above suggest that PTM is affected, however less so, in TssA than it is elsewhere.

The fractional composition shows us that the IP product distribution is reshaped by A485, which in turn suggests that the H3K18ac antibody is still preferentially binding chromatin fragments with TssA annotations. The fraction of IP coming from TssA annotations increased after A485 while it decreased at Quies. This suggests that there is either residual H3K18ac in these annotations or there is a significant off-target species recognized by the H3K18ac antibody. We did not observe drastic reshaping for the H3K27ac antibody.

To summarize the whole genome analysis based on the histogram of annotations ($f(a_i)$), which does not involve making genome browser tracks nor calling peaks, p300/CBP inhibition via A485 results in a deep loss of IP mass and capture efficiency for both H3K27ac and H3K18ac antibodies across all annotations. The H3K18ac antibody in particular shows a residual enrichment of TssA annotations after A485 treatment (Fig. 4C), while the overall capture of those annotations is impaired (Fig. 4A,B). The TssA, TssAFlnk and TxFlnk annotations incurred the weakest losses, while Enh (enhancer) annotations were severely impacted according to both antibodies. We note that GCN5/KAT2A is associated with TssA genomic intervals^24^ and has been shown to have H3K18ac and H3K27ac activity *in vitro*^25^. One hypothesis consistent with all of these observations is that GCN5/KAT2A, which is not inhibited by A485, is maintaining some level of H3K18ac/K27ac at TssA, TssAFlnk, and TxFlnk but not at Enh.

Interestingly, the largest single mass component of the IPs are Quies annotations. This annotation responded significantly to A485 inhibition through both antibodies, suggesting p300/CBP is active in these regions of the genome. Moreover, the lack of focused p300/CBP activity in Quies is consistent with the hypothesis that these regions act as a sink^26^ for excess p300/CBP activity, allowing the accumulation of a non-functioning reservoir of acetylation for recycling^27^. Inhibition of p300/CBP through the inhibitor CBP30 showed modest mass and efficiency losses but demonstrated no significant reshaping of the IP-product distribution for either antibody. We did not try to improve CBP30 impacts by altering treatment paradigm. It remains to be seen whether CBP30 can drive a response similar to that of A485 with an optimized treatment.

### Whole genome analysis using browser tracks

In this section, we describe how siQ-ChIP tracks are computed, aggregated into a database of peak-wise comparisons, and how this database can be used to quickly obtain whole genome conclusions about the data. The database records all genomic intervals corresponding to track peaks as well as several quantitative attributes. The database itself is not a record of a single track, but rather a record of comparisons between tracks. As discussed above, all tracks are made with the $+1/L_i$ accumulation rule. Details of generating siQ quantified tracks and detecting peaks are given in SI (See SI-Fig. 1, 2, and 3 and associated text).

In general, a most common use of ChIP-seq is to test how the ChIP-seq signal reacts to experimental perturbation. To do this here, we first identify an interval $\mathscr {X}$ in a control track and then investigate that same interval in an experimental track. For our p300/CBP inhibition experiments, this means the DMSO track was taken as a control and either CBP30 or A485 data was taken as the experimental track. For each interval detected in the control track, the area under the signal *s*(*x*) is computed for both control and experimental tracks. SI gives details on building $s(x)=\alpha f(x)/f_{\textrm{in}}(x)$ and identifying the complete set of $\mathscr {X}_i$ generated by IP. The minimal Fréchet distance^28^ between the two tracks on the interval is also computed, which provides a numerical assessment of how similar the two tracks are in shape within the given interval. This shape information is included in the database of peaks, but we do not make much use of it here because the degree to which shape is reproducible is currently unstudied. Application of metrics like the Fréchet distance will allow future study of shape. SI-FIG. 2 illustrates this metric with current data, and all drug treatments are summarized in SI-Fig. 3.

The final database is a list of the intervals, the area under *s*(*x*) on the interval for experimental and control tracks, the difference in shape between the tracks, and a few other attributes as noted in the documentation of our tools^14^. Once this database is built, several modes of analyses parallel to those shown in Fig. 4 are possible.

A most informative analysis is represented in Fig. 4B, where the siQ-efficiency of capture is shown as a function of genomic annotation. Of central importance in ChIP-seq is how the sequencing signal, namely the peaks, changed due to experimental perturbation. We define the Response on interval $\mathscr {X}_i$ between $s_{\textrm{cntr}}$ and $s_{\textrm{exp}}$, a control and experimental track respectively, as

7 $$\begin{aligned} r_i = \Big (\sum _{x\in \mathscr {X}_i} s_{\textrm{cntr}}(x)\Big )\Big (\sum _{x\in \mathscr {X}_i} s_{\textrm{exp}}(x)\Big )^{-1} \end{aligned}$$

The sums in numerator and denominator represent the area under *s*(*x*) on the interval $\mathscr {X}$. The response quantifies the change in area under a peak upon experimental perturbation.

A whole-genome characterization of this response is possible by looking at the distribution of responses $\mu (r)$. This distribution (unnormalized) is shown for both A485 and CBP30 inhibition in Fig. 5. The x-axis in these Figures, *r*, is the ratio of areas as DMSO:A485 or DMSO:CBP30. The y-axis is the number of peaks that had a response of $r\pm dr/2$ with *dr* a binwidth. The results after CBP30 inhibition are again not striking. This has been evident since the isotherm was determined (Fig. 3). On the other hand, the results characterizing A485 inhibition show not only a strong response but also a bimodality of response.

The total response distribution can be deconvoluted into contributions from the different annotations as

$$\begin{aligned} \mu (r) = \sum _i \mu (r(a_i))\end{aligned}$$

In practice, this amounts to grouping peaks by the annotation they fall on. This deconvolution is shown in Fig. 5. Of particular interest is the distribution of responses for the TssA annotations. For both H3K27ac and H3K18ac, the distribution $\mu (r(\text {TssA}))$ is shifted to the left and has a long tail to the right side. For H3K18ac, the maximum in $\mu (r(\text {TssA}))$ is near $r=2$ and shows that most of these peaks have small changes in area after A485 treatment compared to DMSO. These are peaks that respond weakly to A485. The long right-side tail indicates that there are still many peaks that did respond to A485 and had a loss in area. For H3K27ac, we found the maximum in $\mu (r(\text {TssA}))$ at $r=5$ meaning there is typically a five-fold reduction in area after A485 treatment. This response is smaller than expected (10-fold for H3K18ac and 6-fold for H3K27ac), where the expected response is estimated as the ratio of $\alpha$’s for the two experiments. We conclude that the response is less than expected for TssA annotations in both H3K18ac and H3K27ac, with the response being severly muted in H3K18ac data. Additionally, there is a larger response in shape perturbations for Enh than TssA (SI-Fig. 3).

Figure 5 combined with Fig. 4 indicates which genomic features/annotations respond to perturbation, how significant that response is, and whether there are peaks associated with the response. Thus, without looking at a single browser track, we have completely described the results of p300/CBP inhibition across the entire genome. The shape of signal in the whole genome can be understood through this analysis without ever focusing our attention on a single isolated peak or gene. To connect this abstract and general characterization to the more familiar representation of tracks, Fig. 6 shows a region of the genome where one can appreciate both responsive and non-responsive peaks. One may also appreciate that H3K27ac has a more muted response on TssA in this window, and H3K18ac has nearly no response on TssA in this window. Meanwhile, peaks on Enh annotations are lost. The diversity of peak responses summarized in Fig. 5 are clearly visible even in this small window on chromosome 1.

### Relationship between siQ-efficiency and binding constants

In SI we restate the model that underlies siQ-ChIP^11^. In short, the total amount of each chromatin species (unique epitope) is denoted $S^t_i$ where $i=1,2,...$ is an index for the species. We write $S^b_i$ for the amount of species *i* that is *bound* in the IP. As shown in the SI, we also have $S^b_i = S^t_i\big (\frac{AB^f K_{B,i}}{1+AB^f K_{B,i}}\big )$, where $AB^f$ is the concentration of free antibody and $K_{B,i}$ is the binding constant of the antibody to species *i*. The siQ-ChIP efficiency for species *i* is $e_i = S^b_i/S^t_i$. Combining these equations we see the siQ-efficiency for the species is given by the isotherm for the species $e_i=AB^f K_{B,i}/(1+AB^f K_{B,i})$. The overall spirit of siQ-ChIP is to let the isotherm determine the quantitative scale, as clearly demonstrated here. One can further leverage these results to find

8 $$\begin{aligned} \frac{K_{B,i}}{K_{B,j}} = \frac{\frac{e_i}{1-e_i}}{\frac{e_j}{1-e_j}} \end{aligned}$$

with *i* and *j* being the index of two different species. We have used $AB^f K_{B,i} = \frac{e_i}{1-e_i}$ to reach Eq. (8).

Equation (7) expresses the ratio of binding constants for two species in the IP. We call this ratio a relative binding constant, and it allows us to estimate how different the binding constants are for species *i* and *j*. The difficulty with chromatin is that we can not exactly partition our massess and sequencing reads into contributions from *i* and *j*, but as we’ve shown above we can approximate this partitioning through the use of the 15-state annotations. These partitions contain mixtures of different species and mixtures of chromatin that presents differing degrees of heterogeneity. As such, these binding constants are ‘apparent’ binding constants. In terms of the discussions above, for the annotations we have $e_i = S^b_i/S^t_i=\alpha f_{\textrm{IP}}(a_i)/f_{\textrm{input}}(a_i)$. Figure 7 shows the relative binding constants for the different inhibitor conditions as relative to the heterochromatin binding constant. We use heterochromatin (Het) as a reference for no particular reason, any reference is suitable. This choice, however, allows us to see dynamics in the TssA category where choosing TssA as a reference would push all the dynamics into all other categories (the relative binding constant at TssA would be 1 for all conditions).

Conclusions drawn from Fig. 7, where data are presented as relative binding constants, simply recapitulate what we learned from the siQ-efficiencies in Fig. 4B. For the H3K18ac antibody, the relative binding constant for TssA increases from 10 to 30 times that of the Het annotation. This could indicate a loss of binding capacity in Het annotations or it could indicate an increased binding capacity at TssA. Figure 4B clearly shows a relatively stable capacity in TssA annotations with a loss in Het. The efficiency is absolute and unambiguous, while the relative binding constants are relative. Nonetheless, an advantage of relative binding constants (over absolute binding constants) is that they are accessible without titration. The relative binding constants can be expressed as a function of annotation or particular genomic intervals, for instance peaks in sequencing tracks. Note however that the siQ-efficiency is already revealing trends in the binding constants, in absolute terms, because the efficiency at any peak is expressed as $e_{\textrm{peak}}=AB^fK_{B,\mathrm peak}/(1+AB^fK_{B,\mathrm peak})$. Without needing a titration, and without determining a global binding constant from the isotherms in Fig. 3, different peak heights can be directly understood as differences in ‘apparent’ binding constants for the genomic regions underlying the peaks^29^. The relative $K_B$ between peaks can be estimated by $e_i/e_j \times (1-e_j)/(1-e_i)$, where $e_l$ is the efficiency at peak *l*. Keep in mind that these binding constants are a summary of various contributions to binding and should not be thought of as exact single species binding constants as determined by isothermal titration calorimetry, for example.

### Quantitative scale and ChIP-seq interpretation

Figure 4C shows that there is an increased fraction of TssA associated fragments in the H3K18ac IP after A485 treatment, and Fig. 5 shows that the peaks associated with TssA annotations have an extremely weak (roughly 2-fold) response. siQ-ChIP applies a global scaling factor, $\alpha$, to the sequencing tracks, and the H3K18ac $\alpha$ was decreased 10-fold upon A485 treatment. For peaks in the sequencing track to decrease by only 2-fold while $\alpha$ decreases by 10-fold, peaks in the unscaled track (before $\alpha$ is applied) must have increased in magnitude by 4 to 5-fold. This increase offsets the decrease in $\alpha$, leading to the muted response. This very clearly illustrates the problem of treating unscaled ChIP-seq data as though it were quantitative, where the physical scale of the IP binding reaction is ignored.

Figure 8 shows metaplots for H3K27ac and H3K18ac at TssA and Enh. In light of everything discussed above, the metaplots in unscaled units (indicated as IP/input) should be interpreted as reflecting the ratio of probability of finding fragments at a position (IP to input), not the *amount* of PTM. H3K27ac shows no evidence of redistribution in the (unscaled) metaplots for TssA, consistent with the analysis above. H3K27ac displays a loss in probability on Enh, again consistent with Fig. 4C. We clearly see a large increase in track height at TssA for H3K18ac in unscaled units (Fig. 8B), with little change at Enh. In all cases the scaled version of these plots reflects the significant losses that were indicated in Fig. 4B.

When sequencing tracks are not understood as representing probability densities, an easy misinterpretation is to conclude that there is an increase in H3K18ac at TssA after A485 treatment. Indeed, this conclusion is reported in several papers^17–20^. In one study, the increase in H3K18ac signal at TssA was described as an ‘increase in the average level of H3K18ac and H3K27ac’^30^. In another study, using spike-ins, it was ‘observed that the H3K27ac mark was reduced at [TssA] whereas H3K18ac was slightly increased’^18^. Yet another recent report observed that ‘surprisingly, only 226 [of 807] downregulated genes exhibited hypoacetylation of H3K27 under the condition of CBP/p300 HAT inhibition’^20^. Consistently, misinterpreting changes in probabilities as changes in abundance leads to the conclusion that the targeted acetylation may be unaffected or even increased in some genomic regions — counter to expectations and contrary to the indication of global loss in the isotherms (IP mass capture, Fig. 3), which mirror what is seen by other global measures like western blots (Figure 3a of Reference^30^ and SI-Fig. 4 herein).

The emergent constraint that sequencing tracks must sum to the total depth means that when we normalize to the depth, the resulting track is interpreted as a probability.

Any track unscaled by $\alpha$ that respects the normalization constraint expresses the probability of a random fragment containing a given base pair, for any specified base pair. Thus, for the H3K18ac antibody, increases in peak height only indicate increased probability of sampling fragments associated with TssA in the IP. This increase in probability does not always indicate more PTM at TssA, since for example it could also follow from leaving PTM at TssA fixed and reducing PTM everywhere else in the genome. This situation would drive an increase in the odds of antibody binding to fragments from TssA.

The sequenced tracks are *f*(*x*) in the above discussion, and thus in general are representative of the probabilities of finding coverage of genomic intervals — even if the normalization constraint is not enforced. Without the normalization constraint, however, the shape of *f*(*x*) can become distorted depending on genomic coverage as we demonstrated with the example in Fig. 2. An increased probability is not an indication of an increase in absolute amount of PTM. For the most part, this misinterpretation does not do much harm, aside from the errant notion that the PTM has actually increased, especially when orthogonal data are integrated. However, using the correct physical scale will not only avoid seemingly inconsistent conclusions, it may also help bring ChIP-seq and RNA-seq, along with other orthogonal observations, into better alignment^20^. We speculated above that the homeostatic mechanism^30^ implied by the increased probability of finding H3K18ac fragments at TssA after A485 exposure could be explained by GCN5/KAT2A which will have activity independent of p300/CBP inhibitors. An alternative hypothesis would involve genomic compartmentalization of HDAC enzymes, where perhaps these ‘erasers’ of lysine acetylation are less likely to associate with TssA regions. In either case, there is a pool of H3K18ac that is less perturbed than others and the absolute amount of PTM is not increasing.

## Conclusions

We have described an intuitive simplification of the siQ-ChIP scaling factor $\alpha$, utilized a sensitive ChIP-seq protocol for use with crosslinked samples^12^, and developed a genome-wide pipeline for siQ-ChIP data analysis that allows one to easily visualize the distribution of IP mass across annotation classes, and to investigate the full distribution of responses elicited by any cellular perturbations. We hope these advances improve the acceptance and applicability of the siQ-ChIP quantitative method.

Additionally, our protocol gives a simple and controlled distribution of fragment lengths because we use MNase rather than sonication. This improves the accuracy of any mass-to-concentration units conversions where the average fragment length is used.

We have also shown there is a strict condition on how the sequenced data can be used, a condition forbidding over-counting of fragments. This may seem like a minor point but it leads to the interpretation of siQ-ChIP data as a mass distribution, which shows how mass, or concentration of captured species, are distributed along the target genome. Moreover, this simple change to track building precludes the common misinterpretation of ChIP-seq data by mandating that tracks be regarded as proportional to probabilities not PTM abundances. Given numerous schemes that have been developed to normalize sequenced data, our proposal to avoid over-counting and normalize solely to the depth to yield $\sum _x f(x)/\hat{R} = 1$ is novel. The constraint on overcounting and the interpretation of quantified ChIP-seq as a ‘mass distribution’ make a compelling argument for the soundness of the siQ-ChIP method and suggests an incompleteness of other quantification schemes.

It is worth discussing that even after $\alpha$ and the siQ-ChIP protocol are streamlined, some practitioners will not find this approach to be easier than standard ChIP-seq. Moving from qualitative to quantitative experimentation does require additional diligence and care, and there is a learning curve. Because siQ-ChIP is quantitative, there are several practical details that should be mastered and validated. These details include checking that antibody is captured on the magnetic beads used in the IP, minimizing complexity in the chromatin fragmetation products, minimizing bead-only capture in the absence of antibody without invoking any additional practices of bead blocking or pre-clearing. Not to mention, siQ-ChIP dictates that IP conditions be matched and we provide one method of doing just that. Interestingly, even if one uses spike-ins for ChIP-seq the IP conditions should be matched. Yet, no evidence or mention of matching is ever included in publication. The protocol used here covers all of these concerns and represents a significant step toward standardizing a quantitative ChIP-seq practice. Further details of the protocol itself are available elsewhere^12^.

## Materials and methods

### Cell culture and drug treatment

HeLa cells (ATCC #CCL-2) were maintained in DMEM (Gibco, 11965092) supplemented with 10% FBS (Sigma, F0926) and 1% penicillin-streptomycin (Gibco, 15140122) and were grown in 37 $^{\circ }$C with 5% CO$_{2}$. Cells were passaged and plated 1 d before drug treatment. Media was then removed and replaced for drug treatment with 10 µM CBP30 (Cayman #14469 Batch: 0473336-73), 10 µM A485 (Cayman #24119 Batch: 0581192-13), or vehicle dimethyl sulfoxide (DMSO) (Sigma 472301, Lot: SHBK2080). DMSO was volume matched for experiments and was either at 0.1% or 0.02% total volume. Cells were treated for 16 h and were then fixed and collected using the protocol described herein.

### ChIP

Cell fixation and collection The volumes listed were for a 10 cm dish of HeLa cells at approximately 70% confluency. Cells were rinsed once with 10 mL of D-PBS (Gibco, 14190136) followed by cross-linking for 5 min in 10 mL of 0.75% formaldehyde (Pierce, 28906) in D-PBS at room temperature. Formaldehyde was removed, and cells were quenched for 5 minutes by addition of 10 mL of 750 mM Tris. Cells were washed twice with 10 mL of D-PBS, scraped into cold D-PBS, collected by centrifugation at 300 g, and snap-frozen in liquid nitrogen. At this point, cells were stored at $-80\,^{\circ }$C.

Chromatin Isolation Cells were then lysed under hypotonic conditions in 1 mL of 20 mM Tris-HCl pH 8, 85 mM KCl, 0.5% NP- 40 (1 tablet of protease inhibitor (Roche, 11836170001) per 5 mL of buffer) for 30 min on ice. Nuclei (and other insoluble material) were collected by centrifugation at 1300 x g for 5 min at 4 $^{\circ }$C, lysed by resuspension in 150 µL 50 mM Tris-HCl pH 8, 150 mM NaCl, 2 mM EDTA, 1% NP-40, 0.5% sodium deoxycholate, 0.1% SDS (1 tablet of protease inhibitor per 5 mL of buffer), and passaged five times through a 27-gauge needle (BD #309623 Lot: 0227218). Lysate was then diluted to 500 µL by addition of 350 µL of binding buffer (25 mM HEPES pH 7.5, 100 mM NaCl, 0.1% NP-40). Five µL of RNAse A/T1 (Thermo Scientific, EN0551) was added, and the sample was incubated at 37 $^{\circ }$C for 25 min. Next, CaCl_2_ was added to a final concentration of 40 mM (21 µL of 1 M) followed by the addition of 75 U (3 µL of 25 U/µL) of micrococcal nuclease (MNase, Worthington Biochemical) and incubated at 37 $^{\circ }$C for 5 min. MNase was quenched by the addition of 40 mM EDTA (46 µL of 500 mM EDTA), and the total volume was brought to 1 mL by the addition of 425 µL of binding buffer. Next, insolubilities were removed by centrifugation at max speed (about 21,000 x g) at 4 $^{\circ }$C for 5 min, and the supernatant containing soluble chromatin was collected.

Chromatin Measurement At this stage, 5 µL of chromatin was measured using the Qubit dsDNA HS Assay Kit (Invitrogen, Q32851). To ensure similar chromatin concentrations and match IP conditions, samples were diluted with binding buffer to match each other.

Antibody to bead conjugation For each IP, 25 µL of Protein A coated magnetic beads (Invitrogen, 10008D) were washed once with binding buffer and incubated with either 0, 1.6, 2.5, or 10 µL of antibody against the target histone mark. Total volume of bead+antibody was brought to 200 µL using binding buffer and were rotated at room temperature for 15 min. Buffer containing antibody was removed, and beads+antibody were resuspended in 200 µL of soluble chromatin followed by 15 min rotation at room temperature. Fifty µL of chromatin was set aside for input. Unbound chromatin was removed, and beads were vortexed for 10 s with 500 µL of binding buffer. Buffer was removed, and bound material was eluted from beads by vortexing for 10 s in 133 µL of elution buffer (25 mM HEPES pH 7.5, 100 mM NaCl, 1% SDS, and 0.1% NP-40). At this time, the input was brought to 133 µL by the addition of 83 µL elution buffer. Proteins were digested by the addition of proteinase K (Invitrogen, 25530015) to a final concentration of 15 µM overnight at 37 $^{\circ }$C. The following morning, each DNA sample was purified using MinElute PCR Kit (Qiagen, 28004) and eluted in 30 µL of Buffer EB. Five µL of DNA was quantified by Qubit dsDNA HS Assay Kit. The remaining 25 µL of DNA was frozen at $20\,^{\circ }$C until it was prepared for sequencing libraries. For comparison of a mark between samples, we performed ChIP of all samples on the same day and made a master mix of bead+antibody for each ChIP target, scaling up all components by the number of samples.

DNA gel DNA fragment size of inputs was checked on 1X TBE 2.5% agarose gels with 1X SYBR Safe (Invitrogen, S33102) to ensure MNase digestion. One µL of NEB ladder (#N3231S Lot: 10047328), run at 60 V for 60 min.

### Library preparation and sequencing

Details such as the amount of DNA taken into library preparation can be found in Supplementary Table 1. Library preparation was done using KAPA HyperPrep Kit (Roche, KK8504) with 4 µL of Illumina adapters (IDT, UDI) and sequenced on Illumina NextSeq 500 with a Mid-Output (paired-end 75 bp reads) flow cell. Input libraries had between 83-102M reads, and each IP library had 27-46M reads that passed QC, with 88% of the bases having quality scores $\ge$30.

### Antibodies list

For ChIP-seq

H3K27ac (Active Motif, 39133 Lot 06921014–Fig. 3), biological replicates from Supplementary Figure SI-Fig. 5A use lots 06921014 and 16119013

H3K18ac (Active Motif, 39755 Lot 26919002) Fig. 3

H3K18ac (Invitrogen, MA5-24669 Lot: WB3 187272) Supplementary Figure SI-Fig 5B

For Western Blot SI-Fig. 4

H3K27ac (Active Motif, 39133 Lot 16119013) 1:2000

H3K18ac (Invitrogen, MA5-24669 Lot: WB3 187272) 1:2000

Total H3 (Epicypher, 13-0001 Lot:12320001) 1:50000

Rabbit Secondary (Cytiva, NA934V Lot: 17016966) 1:10000

### NGS data processing

We followed exactly the same procedure as previously described^11^.

## Supplementary Information

Supplementary Information.

## Data Availability

All codes for analysis and figures are available at GitHub^14^, as are the full database files for all peaks in Fig. 5. (database: https://github.com/BradleyDickson/siQ-ChIP/blob/master/databases-p300.tgz) The genomics data are available via GEO code GSE207783.
