# Supplementary material for: Streamlined quantitative analysis of histone modification abundance at nucleosome-scale resolution with siQ-ChIP version 2.0
Source: Sci Rep. 2023 May 9;13:7508. doi: 10.1038/s41598-023-34430-2 (PMC10169836; doi:10.1038/s41598-023-34430-2)
Supplement: Supplementary file 1 — Supplementary Information. [file 41598_2023_34430_MOESM1_ESM.pdf]

Supplementary Information: Streamlined quantitative analysis of histone modification abundance at nucleosome-scale resolution with siQ-ChIP version 2.0

Bradley M. Dickson, Ariana Kupai, Robert M. Vaughan and Scott B. Rothbar[1, *]

[1] vari

(Dated: March 29, 2023)

Keywords:

ALTERNATE DERIVATION OF α

The siQ-ChIP scale α can be obtained as a units conversion applied to the IP reaction efficiency as follows. The heart of siQ-ChIP is the realization that the IP is subject to the basic mass conservation laws that govern all reversible binding reactions. Namely, the total antibody concentration is equal to the sum of the free antibody and bound antibody concentrations. Because of this, the IP mass must follow a sigmoidal form, where increasing antibody concentration causes increased IP mass up until the reaction is saturated. As we explain next, the work of siQ-ChIP is concerned with two features: the determination of the isotherm and the units conversion that maps IP mass to concentration of antibody-chromatin complex. The concentration of complex is what sets the quantitative scale for siQ-ChIP.

In more formal terms, the sum of free antibody (AB^f) and bound antibody takes the following form

$$AB^t = AB^f + AB^f \sum_{i=1}^{N} K_{B,i} S_i^f \tag{1}$$

where we used the traditional binding constant definition $K_{B,i} = [AB \cdot S_i]/AB^f S_i^f$. S_i is the i-th species or epitope that interacts with the antibody and $[AB \cdot S_i]$ is the concentration of complex. The total antibody mass is also subject to a conservation of mass constraint for each species, $S_i^t = S_i^f + AB^f K_{B,i} S_i^f$ where S_i^t is the total concentration of species i. S_i^f is the free (or unbound) concentration of species i.

The symbol S_i^t represents the concentration of a chromatin 'state'. Without trying to enumerate all possibilities, these could include all mono-nucleosome fragments that present a defined set of histone modifications. There may be another species S_j^t for the di-nucleosome fragments that present the same modifications. Yet another term, S_k^t, for mono-nucleosomes presenting different modifications or combinations of modifications, and so on.

Of interest here is the solution to these mass conservation laws. The solution is just the set of values S_i^f and AB^f that would simultaneously satisfy all of the conservation equations. If we knew the binding constants $K_{B,i}$ then we could generate the solution numerically. Of course, we do not know the binding constants and we also don't know how to enumerate all of the terms in the conservation laws, but we have a very handy way to make these shortcomings moot: We determine the actual IP mass empirically, which is itself the sum of all the bound fragments whatever they are and however they came to be there. We can empirically determine this correct mass without needing to know all the terms and constants exactly.

Formally, we have the total bound concentration of chromatin $S^b = \sum_i S_i^b$ (likewise $S^t = \sum_i S_i^t$), which for our model can be expressed as $S^b = \sum_i S_i^t \left(\frac{AB^f K_{B,i}}{1+AB^f K_{B,i}} \right)$ where we used the bound concentration $S_i^t - S_i^f$. The total S^b is the sum of sigmoids thus, as described above, we anticipate that S^b will plateau or saturate when AB^t is increased.

The key for siQ-ChIP is that this concentration S^b can be converted to mass using the average molecular weight per base pair (660 g/mol/bp) and the average fragment length L, yielding $m_{IP} = S^b(V - v_{in})660L$ which is the IP mass. The factor $(V - v_{in})660L$ converts from concentration units to mass units. Now, once m_{IP} is determined empirically S^b can be estimated using this unit conversion. We can do a similar thing with the determined input mass, $m_{input} = v_{in}660L_{in}S^t$ where S^t is the total chromatin concentration. The quantitative scale put forward by siQ-ChIP is based on the fact that the total IP capture efficiency can be expressed as $\frac{S^b}{S^t} = \frac{m_{IP}}{m_{input}} \times \frac{L_{in}}{L} \frac{v_{in}}{(V - v_{in})}$.

Because some of the IP and input masses will be sequenced, we have knowledge of the genomic coordinates for a representative collection of the chromatin fragments. Using x to denote genomic coordinates and $f'(x)$ to denote any proper summary of the sequenced fragments (*e.g.,* $|f'| = \sum_x f'(x) = \hat{R}$ with $\hat{R}$ the sequencing depth), we can state that $|\frac{m_{IP} \times f'}{(V - v_{in})660L\hat{R}}| = S^b$. We say *proper* here because $|f'| = \hat{R}$ implies that no fragment can be counted more than once. This places a strict constraint on how sequencing tracks are built and interpreted. Typical practice will over count sequenced fragments, with each fragment counted once for each base pair in the fragment. The key result here is that for $m_{IP} f'(x)$ the conversion factor $((V - v_{in})660L\hat{R})^{-1}$ projects $m_{IP} f'(x)$ to $S^b(x)$, which is an estimate of the concentration of bound fragments at x.

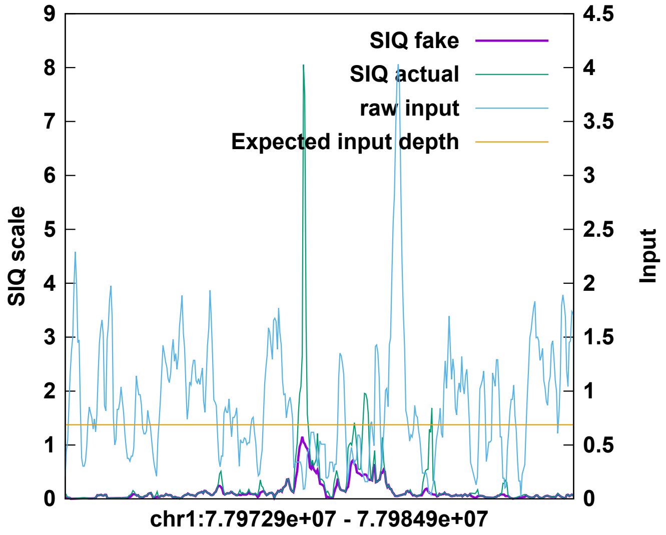

SI-Fig. 1: The impacts of using expected input, or 'fake input', to regularize siQ-scaling.

PROCESSING SEQUENCING DATA

The siQ-ChIP scale is built on the IP to input ratio because it expresses efficiency of capture. Ultimately, this leaves us to evaluate $\alpha f_{IP}(x)/f_{in}(x)$ and to deal with the inevitable case that $f_{in}(x) \sim 0$ while $f_{IP}(x) > 0$. In these cases the IP demonstrates that the genomic region represented by x was present in the chromatin but for statistical reasons has not been presented in the input sequence data.

Because the sequenced input fragments are expected to be binomially distributed along the genome, we estimate the average expected depth, $\langle d\rangle$, of input at any position x as $\langle d\rangle = \hat{R}_{in}p/(1-p)$ where p is the probablity of hitting any base pair in the genome. In our case we use bins larger than a single base pair and p is adjusted to this width. ($p = 30/3200000000$ for bins of 30 base pair and a total of 3200000000 bases.) Any time $f_{in}(x) < \langle d\rangle$ we replace the input with $\langle d\rangle$. We refer to this replacement as 'fake input' and an example of how this impacts data is shown in SI-Fig 1. The siQ-scale should not be larger than unity for any reason other than noise in the determination of α. SI-Fig 1 shows how using the 'fake input' resolves the over unity problem, where it results from sampling errors in the input track. Over unity peaks are still possible, but are less likely.

The siQ-ChIP sequencing track is given by $s(x) = \alpha f_{IP}(x)/f_{in}(x)$. To call peaks in $s(x)$ we first compute

$$\begin{aligned} \langle s\rangle &= \sum_x s(x)/\sum_x 1 \\ \langle s^2\rangle &= \sum_x s^2(x)/\sum_x 1 \qquad (2) \\ \sigma^2 &= \langle s^2\rangle - \langle s\rangle \end{aligned}$$

Any genomic interval $\mathcal{X}$ that has signal satisfying $s(x) > \langle s\rangle$ for all $x \in \mathcal{X}$ and $s(x) > \langle s\rangle + 3\sigma$ for some $x \in \mathcal{X}$ is understood as displaying a peak. This is a simple choice for selecting intervals that have signal larger than apparent background, we did not experiment with values other than 3σ but one can set this value in the siQ-ChIP scripts.

As noted in the Main text, part of the database of peaks includes the Fréchet distance between control and experimental data. The Fréchet distance is a metric of shape-similarity between a peak in the control track and the experimental track. This similarity is computed for each interval $\mathcal{X}$, where the tracks on the interval are mapped to the unit square. We map to the unit square so that there is no unit based disparity between the x- and y-coordinates of the tracks and so that the notion of shape is independent of the height of the peaks. To appreciate the quantitative shape comparison, one can imagine the unit square as a visual display, like a projector screen, comprised of pixels. If the control and experimental tracks are ploted on the display, $(dF)^{-2}$ gives us an idea of the most pixels the display can have while still allowing the two curves to look similar by eye. A large number of pixels implies a high resolution match, corresponding to a small dF value. Conversely, low resolution matches have large values of dF.

For example, a value of $dF = 0.2$ gives us 25 pixels while a distance of 0.4 gives us a 6 pixel display. This small displacement of 0.2 in the value of dF generates a 4-fold reduction the effective resolution for comparing the data. As a rough guide, values smaller than 0.3 will be generally agreed upon as looking similar where larger values will not. In SI-Fig 2 we illustrate how the metric looks for several actual peak comparisons.

The extent to which peak shapes ought to be conserved between samples or treatments has not been quantitatively characterized. SI-Fig 3 reports all the shape response distributions. We point out that the units and scale of the Fréchet metric take some getting used to. To help calibrate to the scale of dF, SI-Fig. 2 reports on a few values of dF.

SI-Fig. 4 reports our global observations of histone acetylation after p300/CBP inhibition. Global losses are clearly reported for A485 while little can be appreciated for effects of CBP30.

SI-Fig. 5 reports on a biological repeat of the isotherms for chromatin:antibody reactoins and reports all bead-only capture amounts. No bead-blocking or preclearing is used, and almost all bead-only capture masses are below 1% by mass.

* Electronic address: bradley.dickson@vai.org

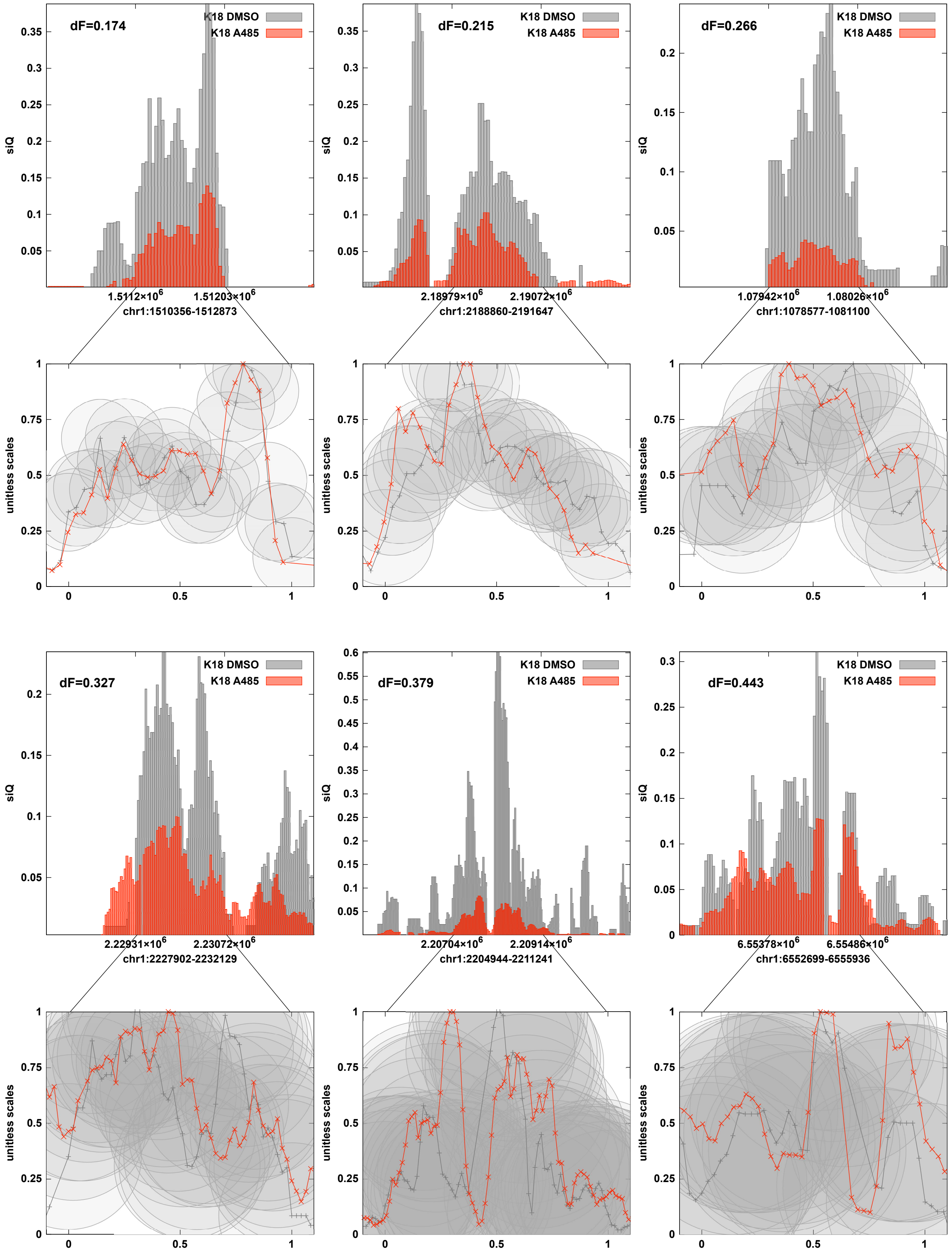

SI-Fig. 2: Examples of peaks in a comparison of DMSO and A485 tracks with the K18ac antibody. The peaks are projected to a unitless rectangle and the Fréchet distance is computed. The circles have radii matching the Fréchet distance dF. The interval containing a peak in the control track is marked by tics on the top of each bar graph and is expanded to the unitless rectangle where the Fréchet distance is illustrated. The region called to contain a peak runs from x=0 to x=1, in each case, as indicated.

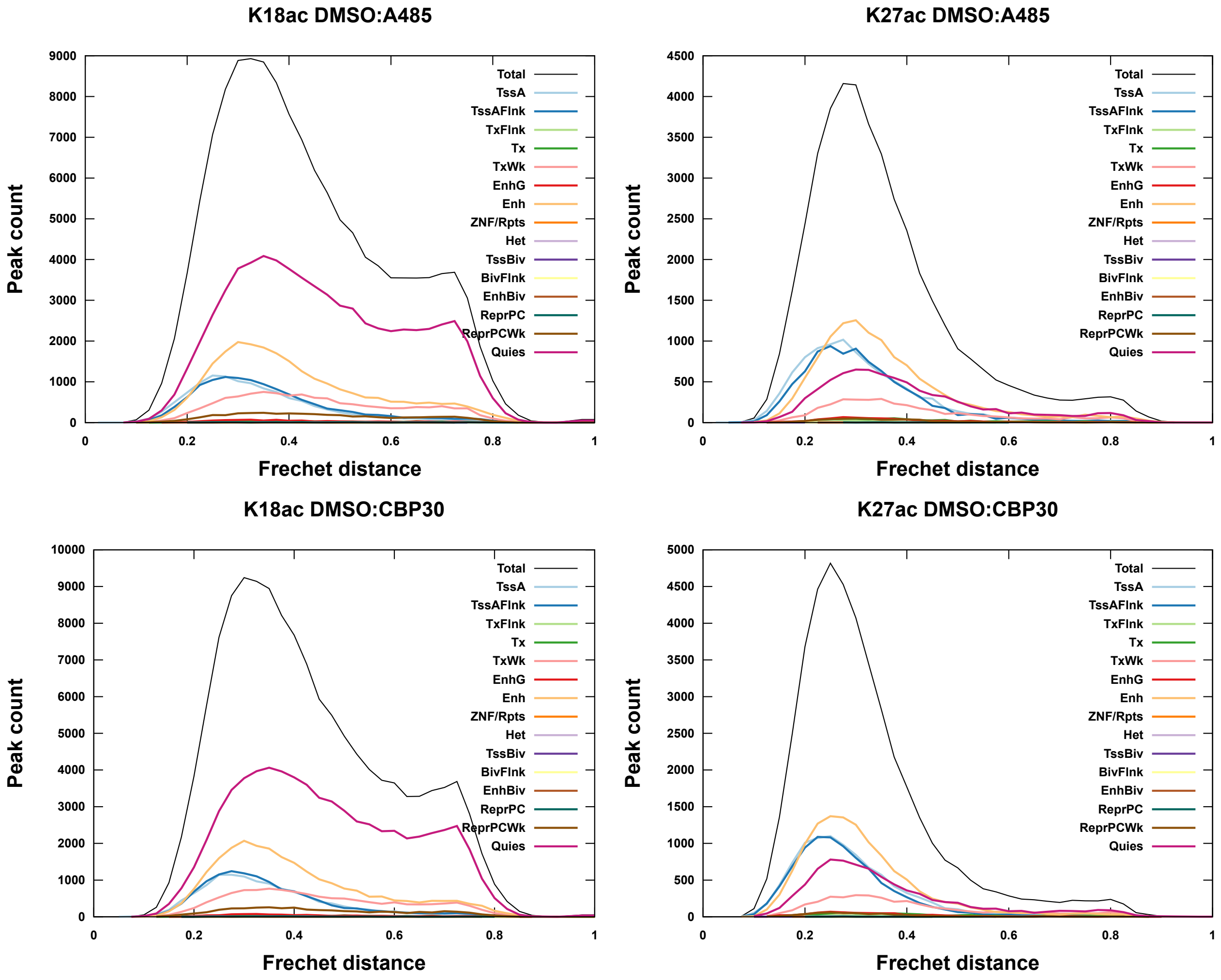

SI-Fig. 3: Fréchet response distributions for all drug treatments.

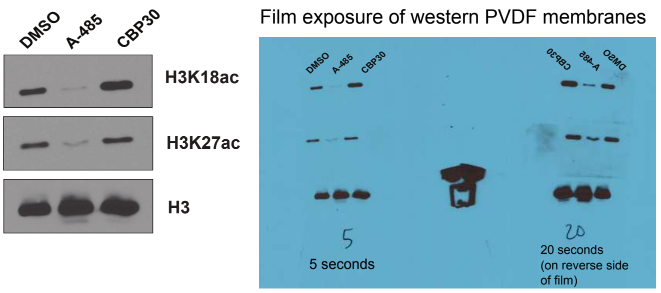

SI-Fig. 4: Western blot for H3K18ac or H3K27ac in treated HeLa cells. HeLa cells treated with 10μM of drug A-485 or CBP30 for 16 hrs. DMSO is volume matched and makes up 0.1% of the total. 2.5μg of chromatin is loaded. Two exposures are shown, 5 and 20 sec (on reverse side of film). Image on the left is from 5 sec exposure cropped to display roughly 10-20 kD.

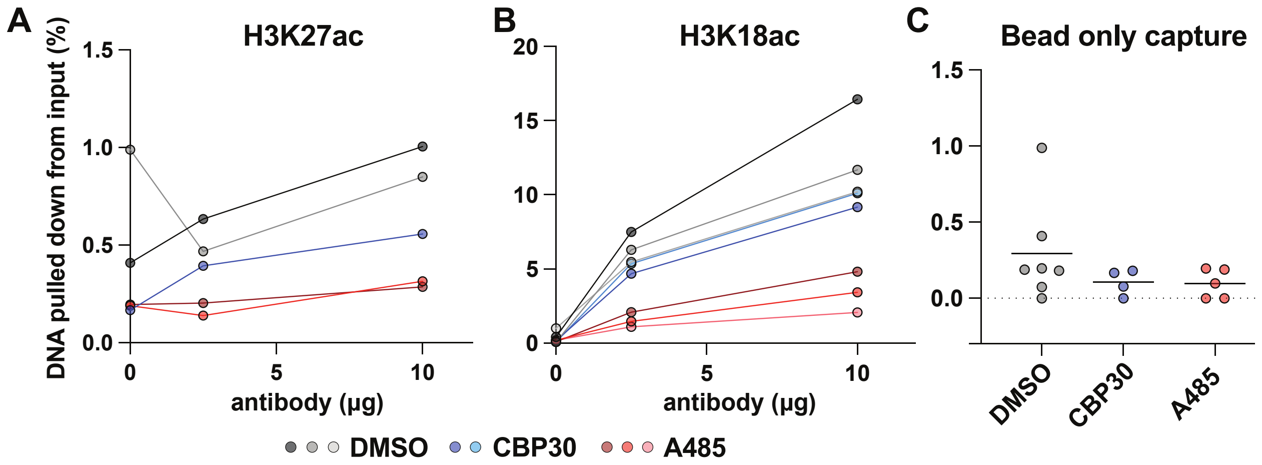

SI-Fig. 5: Biological repeats of (A) H3K27ac isotherms, (B) H3K18ac isotherms, and (C) bead-only capture as percent mass. Note that this H3K18ac repeat is with antibody: Invitrogen, MA5-24669 Lot: WB3 187272

Symbol	Definition	Measurement	DMSOK18ac	DMSOK27ac	CBPK18ac	CBPK27ac	A485K18ac	A485K27ac	DMSOK18ac	DMSOK27ac	CBPK18ac	CBPK27ac	A485K18ac	A485K27ac
$\mathcal{F}$	Fraction of IP taken to library	Total IP mass (ng)	22.68	3.81	16.8	2.052	3.72	0.78	0.2205	0.6667	0.2976	0.6667	0.6667	0.6667
		IP mass into library (ng)	5	2.54	5	1.368	2.48	0.52						
$\mathcal{F}_{in}$	Fraction of input taken to library	Total input mass (ng)	98.7	98.7	102.9	102.9	114.3	114.3	0.0507	0.0507	0.0486	0.0486	0.0437	0.0437
		Input mass into library (ng)	5	5	5	5	5	5						
$\mathcal{F}^{\mathcal{L}}$	Fraction of IP library sequenced	IP library conc. (nM)	144.2	150.2	151.9	136.7	137.2	76.4	0.0055	0.0053	0.0053	0.0059	0.0058	0.0105
		Total IP library volume (µL)	20	20	20	20	20	20						
		Normalized IP library conc (nM)	8	8	8	8	8	8						
		Volume of normalized IP library loaded into sequencer (µL)	2	2	2	2	2	2						
$\mathcal{F}_{in}^{\mathcal{L}}$	Fraction of input library sequenced	Input library conc. (nM)	183.5	183.5	167.8	167.8	190.6	190.6	0.0087	0.0087	0.0095	0.0095	0.0084	0.0084
		Total input library volume (µL)	20	20	20	20	20	20						
		Normalized input library conc. (nM)	8	8	8	8	8	8						
		Volume of normalized input library loaded into sequencer (µL)	4	4	4	4	4	4						
$v_i/(V-v_i)$	Fraction of chromatin taken for input	Volume taken for input (µL)	50	50	50	50	50	50	0.2500	0.2500	0.2500	0.2500	0.2500	0.2500
		Total volume of chromatin (µL)	250	250	250	250	250	250						
ρ	Ratio of captured IP [library] to expected [library]	IP mass into library (ng)	5	2.54	5	1.368	2.48	0.52	0.0464	0.0824	0.0431	0.1279	0.0795	0.1852
		PCR amplifications (cycles)	12	12	12	12	12	12						
		Total IP library volume (µL)	20	20	20	20	20	20						
		IP library conc. (nM)	144.2	150.2	151.9	136.7	137.2	76.4						
		Average IP fragment length (bp)	499	432	440	397	446	391						
ρ_{in}	Ratio of captured input [library] to expected [library]	Input mass into library (ng)	5	5	5	5	5	5	0.0452	0.0452	0.0384	0.0384	0.0438	0.0438
		PCR amplifications (cycles)	12	12	12	12	12	12						
		Total input library volume (µL)	20	20	20	20	20	20						
		Input library concentration (ng/uL)	183.5	183.5	167.8	167.8	190.6	190.6						
		Average input fragment length (bp)	382	382	355	355	357	357						
α	$\alpha = v_{in}/(V-v_{in})\ \rho_{in}/\rho\ (\mathcal{F}_{in}^{\mathcal{L}})/\mathcal{F}^{\mathcal{L}}\ \mathcal{F}_{in}/\mathcal{F}$	The proportionality constant that maintains connection between IP'd material and sequencing reads (fragments)	0.08795463	0.017067	0.065863	0.008916	0.0130257	0.0031154	0.0880	0.0171	0.0659	0.0089	0.0130	0.0031

SI Table 1: All siQ ChIP Measurements
